# Sodium benzoate and rifaximin are able to restore blood-brain barrier integrity in he cirrhotic rats

**DOI:** 10.1186/2197-425X-3-S1-A691

**Published:** 2015-10-01

**Authors:** D Thabut, S Mouri, H El Mourabit, R Morichon, D Wandum, E Lasnier, C Housset, N Weiss, Brain Liver Pitie-Salpetriere Study Group (BLIPS)

**Affiliations:** Assistance Publique - Hopitaux de Paris, La Pitié-Salpetriere Hospital, Hepatological ICU, Paris, France; Assistance Publique - Hopitaux de Paris, La Pitié-Salpetriere Hospital, Paris, France; Neurology Department, Assistance Publique - Hopitaux de Paris, La Pitié-Salpetriere Hospital, Neurological ICU, Paris, France

## Introduction

Hepatic encephalopathy (HE) is a severe complication of cirrhosis which independently influences prognosis. We previously showed an increase in blood-brain barrier (BBB) permeability in cirrhotic rats with HE.

## Objectives

The aim of the present work was to assess the effects of sodium benzoate (Bna), a drug removing ammonia through non-urea cycle pathway, and Rifaximin (RFX), a non adsorbable antibiotic, on BBB permeability in cirrhotic rats with HE.

## Methods

Three groups of rats were considered: SHAM, Bile Duct Ligation (BDL), BDL + hyperammonic dietary (BDL-NH3). In each group, rats were treated by BNa or RFX. HE was assessed using neurocomportemental testing (6 minutes tail suspension test assessing the time of immobility). NH3 levels were assessed before sacrifice. BBB permeability was assessed by IV injection of a fluorochrome (Texas Red 10kDa) before transcardial washing. Brain fluorescence was estimated by fluorimetry after right hemisphere squeezing.

## Results

Mean time of immobility was longer in BDL-NH3 and BDL rats than in SHAM (p = 0.0004). Ammonemia was significantly higher in the BDL-NH3 than in BDL rats, and higher in the BDL than in SHAM rats (p < 0.0001). Intra-cerebral fluorescence was significantly higher in BDL-NH3 than in BDL group, and higher in BDL than in SHAM group (p = 0.029) confirming the passage of the fluorochrome through the BBB. BNa treatment significantly decreased ammonemia levels and intra-cerebral fluorescence in the BDL and BDL-NH3 rats (p < 0.04 for all) but did not modify the mean time of immobility. On the contrary, RFX treatment did not modify ammonemia levels but significantly decreased intra-cerebral fluorescence (p < 0.05) and the mean time of immobility (p = 0.0004).

## Conclusions

In cirrhotic rats displaying HE, BBB permeability is increased, through different mechanisms dependent and independent of hyperammonemia. BNa and RFX are effective in restoring BBB integrity in HE cirrhotic rats but only RFX is able to decrease HE in this model.

